# Establishing Cell Models to Understand Cellular Toxicity: Lessons Learned from an Unconventional Cell Type

**DOI:** 10.3390/toxins14010054

**Published:** 2022-01-12

**Authors:** Tino Vollmer, Bernd Stegmayr

**Affiliations:** 1 Department of Internal Medicine I, Medical Center—University of Freiburg, Faculty of Medicine, University of Freiburg, D-79106 Freiburg, Germany; 2 Department of Public Health and Clinical Medicine, University of Umea, SE-90187 Umea, Sweden; bernd.stegmayr@umu.se

**Keywords:** cell models, cellular toxicity, uremic toxins, chronic kidney disease, hemodialysis

## Abstract

The syndrome of uremic toxicity comprises a complex toxic milieu in-vivo, as numerous uremic substances accumulate and harm the organ systems. Among these substances, toxic and non-toxic players differently interfere with human cells. However, results from animal experiments are not always compatible with the expected reactions in human patients and studies on one organ system are limited in capturing the complexity of the uremic situation. In this narrative review, we present aspects relevant for cellular toxicity research based on our previous establishment of a human spermatozoa-based cell model, as follows: (i) applicability to compare the effects of more than 100 uremic substances, (ii) detection of the protective effects of uremic substances by the cellular responses towards the uremic milieu, (iii) inclusion of the drug milieu for cellular function, and (iv) transferability for clinical application, e.g., hemodialysis. Our technique allows the estimation of cell viability, vitality, and physiological state, not only restricted to acute or chronic kidney toxicity but also for other conditions, such as intoxications of unknown substances. The cellular models can clarify molecular mechanisms of action of toxins related to human physiology and therapy. Identification of uremic toxins retained during acute and chronic kidney injury enables further research on the removal or degradation of such products.

## 1. Introduction

Human cell models must be developed alongside the disease of interest. The syndrome of uremic toxicity includes complex variables to be considered for creating in-vitro conditions adequate to the uremic state. During acute and chronic kidney failure, numerous substances are retained in higher concentrations in uremic patients and are denominated as uremic toxins [1,2]. It is noteworthy that more uremic substances are likely to be identified [3] and some substances may also induce non-toxic or even protective cellular effects. A cellular model of uremic toxicity has to be flexible with regards to the number of uremic substances and the physiological readout covering the toxic and protective uremic effects.

Uremic patients suffer from various and different symptoms depending on the degree of toxicity, but also on individual pathological reactions and responses to such substances. A negative effect of various toxins may be identified by various cell models or exposure of substances to animals [4]. However, results from animal experiments are not always compatible with the expected reactions in human patients. Therefore, additional analyses using human methods are important in order to explore physiology.

The exposure of specific toxins to humans meets ethical limitations. However, such exposure was made in 1972 when urea was added to the dialysate in patients with far-advanced renal failure. There were no major toxicity findings noted in that study if the dialysate concentration was below 30 mmol/L, while the fast removal by dialysis from these levels caused side effects [5].

Mechanistic studies, using cell models derived from one organ system, can identify and characterize single uremic toxins; however, they are naturally limited in capturing the complexity of the uremic situation. The uremic situation comprises a dynamic environment that is altered by the physiological response of the cells, their pathological decay, and the clinical situation of the patients, e.g., their medication. Our team developed a human spermatozoa-based model for the evaluation of toxic effects on cell viability and cell motility [6]. Spermatozoa are highly differentiated and unique cells, e.g., protein biosynthesis is suppressed in spermatozoa and hence, transcriptional and translational activity are vastly reduced [7]. In contrast, post-translational processes are critically involved in the activation of spermatozoa and the development of their fertilization capacity to execute progressive motility [8]. Proteomic studies have identified a significant proportion of proteins in spermatozoa that are associated with the metabolism and energy production [9]. Besides anaerobic glycolysis, oxidative phosphorylation (OXPHOS) in the mitochondria of spermatozoa plays a crucial role in the energy supply for progressive motility [10,11]. Hence, progressive motility of spermatozoa depends on mitochondrial function. Intriguingly, mitochondrial toxicity has been reported to be an elementary mode of action, by which substances, pollutants, and drugs can influence the physiological functions [12,13,14,15], for example multiple drugs have been shown to interfere with the distinct pathways relevant for mitochondrial equilibrium [16,17]. The use of the progressive motility of animal-derived spermatozoa as a method to identify the toxic effects of substances has been previously implemented [18,19,20]. The toxic effects by substances on human spermatozoa motility have been likewise reported [21,22,23,24]. However, the availability of robust human motility test systems that allow for a broad and serial substance evaluation for putative toxic effects on mitochondrial function is limited.

Our human model combines the analysis of apoptotic and mitochondrial cell function. Firstly, our model includes a visual cell death evaluation resembling a putative expression of cell membrane stability in an analogy to models evaluating the median lethal dose (LD50). Secondly, our model includes a cell motility evaluation resembling putative interference of motility patterns and myofibrillar functions. The integration of cellular viability with its functional response allows for a more stringent resemblance of the uremic toxicity situation.

**Figure 1:**
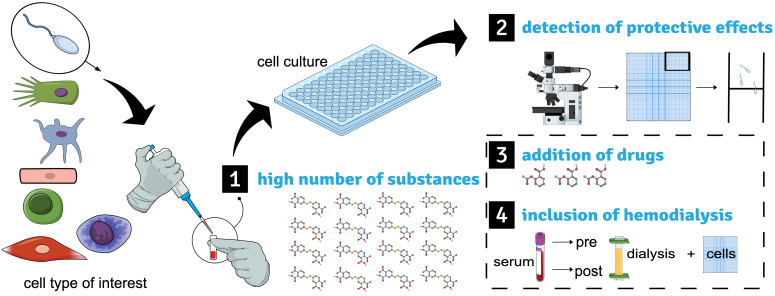
Graphical abstract on establishing cell models to understand uremic toxicity. The human spermatozoa-based model is shown as an example as previously established [6]. The cell type of interest (here spermatozoa) is incubated with uremic retention solutes in a sufficiently high number in single conditions (**1**). The cellular motility analysis allows us to detect the protective effects of the substances according to the previously established method (**2**). Drugs can be likewise applied to the in-vitro setting and complement the uremic setting (**3**). The back-to-back comparison between serum accessed before and after hemodialysis within the cellular readout allows for early implementation of the treatment of interest (**4**).

This narrative review aims to give general guidance for the establishment of cellular toxicity models. In the following review, we present four key aspects based on our previous establishment of a spermatozoa-based cell model that may expand the impact of a cell model to study uremic toxicity (Figure 1).

## 2. Serial Testing of Uremic Substances

The uremic milieu comprises a growing number of retained uremic substances. By the current identification of more than 100 uremic substances [25], the cell model needs to be applicable for the serial testing of substances. Uremic substances can be sub-divided based on their molecular characteristics into the following three classes: small molecules, middle molecules, and protein-bound substances [1]. Uremic toxin studies tend to cover substances within one class based on their underlying biochemically driven research approach. In our study, we observed the toxic effects from substances of all of the three classes and we performed a hierarchical and inter-class comparison of >40 uremic substances. This allows for a more exhaustive picture of the uremic state as opposed to uremic analysis restricted to one molecular class. A major experimental aim of an assay should thereby be its feasibility within a reasonable time and its reproducibility by means of a straightforward experimental set-up. The cell culture assays that exceed 24 h of cultivation may impede this goal. By the application of uremic substances in the highest or maximum concentration reported in uremic patients (C_max_) [3], we observed a rapid change in viability and motility of the cells. In detail, we detected the uremic toxic effects in a time range of 120 min, which allowed for high reproducibility and serial testing of uremic substances. Ultimately, a back-to-back comparison between multiple substances results in a more precise toxic profile of the uremic situation.

## 3. Detection of Protective Effects by Cellular Responses

**Figure 2:**
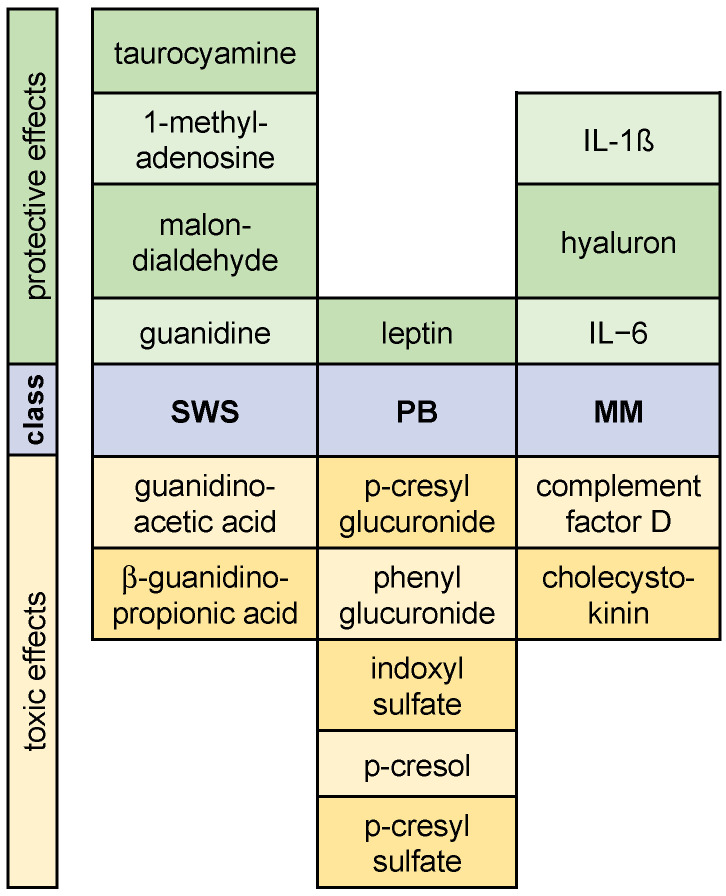
Schematic summary of our study that shows uremic retention solutes detected to induce toxic or protective effects within a total screen of 47 substances [6]. Protective was defined as an absolute increase in motility of >25% (upper panel, green), toxic was defined as an absolute decrease in motility of >25% (lower panel, yellow). Substances are aligned to their molecular class: small water soluble (SWS), protein-bound (PB), or middle molecules (MM) (middle panel).

The distinct uremic retention solutes should be excluded from the removal strategies based on their beneficial role in physiology. It is noteworthy that uremic substances are denominated as uremic *toxins* based on their accumulating state in serum. This accumulation in serum does not necessarily associate with a high degree of cellular toxicity. We reason that experiments should specifically aim to uncover the protective roles of uremic substances. Ideally, a developed cell model can identify a toxic effect but also a putative *protective* effect of a substance. Holistic functional cellular readouts are considerably suitable to characterize cell states beyond two categories, e.g., dead versus alive. The cellular readout analysis comprises a quantitative or “gray” scale and can give more detailed guidance in assessing the effect of a substance in the broad spectrum between *toxic* and supposedly protective. Cellular motility serves this aim since both states of substance-induced hypomotility versus hypermotility represent a functional continuum that indicates the effect of a substance. Following this approach, we could identify uremic retention solutes from all three of the molecular classes that are assigned to the toxic category (as defined by hypomotility) and those that are assigned to the protective category (as defined by hypermotility) (Figure 2).

## 4. Addition of Drugs to the In-Vitro Setting

Drugs can interfere with human physiological function beyond their original targets. This is especially important in the uremic situation as the serum levels of drugs can increase significantly in more progressive pre-dialysis conditions. We reason to incorporate medication conditions into uremic cell models in addition to a significant number of uremic toxins (see 2). In detail, we recommend transferring the methodology of applying uremic substances in C_max_ (the highest concentration observed in a uremic patient) in an analogous manner for drugs. This enables us to investigate the real serum toxicity of patients in pre-dialysis conditions. Furthermore, we suggest including drugs within the identical experimental design of testing uremic substances. In order to estimate the degree of toxicity of a serum-accumulating drug, it is critical to compare it directly to the effect of a uremic toxin.

The described model also allows for the separate investigation of drug toxicity, such as applicability to screen for chemotherapy induced toxic effects. These investigations can also be performed on the biological fluid from patients exposed to cytotoxic drugs prescribed by various indications.

## 5. Translation for Clinical Application

The implementation of a clinical aim into an investigational analysis is central to propel the translation of experimental results into diagnostic and therapeutic application. Uremic toxin research can profoundly contribute to the application and development of hemodialysis procedures when experimental conditions are linked to the real-world clinical state. One applicable step is to apply positive controls within uremic toxin experiments that are based on the uremic state of chronic kidney patients before hemodialysis. In our study, we applied a mixture of uremic toxins in C_max_ as a positive control among multiple testing conditions. The positive control condition resulted in rapid cell death and the cease of physiological motility. Such observed effects can underline the sensitivity of a toxic assay and its applicability to the desired clinical aim of hemodialysis.

Hemodialysis can be directly integrated into the experimental set-up, i.e., a comparison between the state of patients before and after treatment. This enables the ultimate identification of the effects caused by hemodialysis (or its detoxification) and moreover, can reveal whether the hemodialytic procedures may fail to do so in individual patient cases. In our study, we directly compared the serum pre- and post-hemodialysis of chronic kidney patients. Notably, we identified one patient that did not exhibit a loss in serum toxicity after the hemodialytic procedure. Ultimately, experiments that identify non-profiting or outlier patients are valuable. These patients can be of outmost significance to follow-up with mechanistical studies. Does a physiological or clinical attribute of this patient contribute to the observed *non*-response to hemodialysis? Studies on these patients may even help to identify novel uremic toxins.

Experimental uremic studies should aim to develop new hemodialysis parameters. Currently, the calculation of Kt/V_urea_ is used for monitoring the effect of hemodialysis, which does not show good predictive value for toxicity monitoring [26]. We hypothesize that toxicity monitoring based on a single uremic substance will be outperformed by a cellular model that integrates pre- and post-dialysis serum analysis, and which is therefore based on clinically relevant cellular toxicity.

## 6. Conclusions and Future Directions

This commentary article presents aspects based on a human spermatozoa model that can help to create further cell models relevant for the investigation of clinical toxicity, such as in uremia. Overall, we favor to study the uremic milieu with the following parameters: (i) inclusion of a wide number of uremic substances, (ii) selection of a cellular readout that extends toxic versus non-toxic categories, (iii) consideration of the treatment of the patients, i.e., drugs, and (iv) early translation in order to improve hemodialysis. The integration of these steps may result in the creation of a more in-vivo-like uremic environment of a cell model.

Multiple studies report on cell death due to uremic toxicity as a driver for physiological decrease after exposure to toxins [27,28,29]. In line with this, we argue to include a robust and representative experimental model for apoptosis, or cell death, that can be used to investigate for toxicity, such as in samples from uremic patients. We recommend using a cell assay that investigates the complete structure of a cell, i.e., the analysis of the cell membrane. The integrity of the cell is a crucial determinant for its survival. Furthermore, the membrane represents the contact area of the cell with its environment. Assays that include cell membrane analysis can detect cellular leakage or porosity because of contact with toxins. Hence, we argue for cell membrane assays to play a key role in the detection of unknown toxins and drugs.

Fewer models investigate the uremic effects on holistic cellular functions that maintain the physiology of organisms. For example, cellular motility is crucial for various forms of cellular communication, such as immune responses [30] or human reproduction [31]. An incremental part in experimental toxicity research is to integrate a specialized cell type into a cellular assay. In this specific case, the progressive motility of spermatozoa can be observed in real-time by light microscopy [32] and motility is conserved in-vivo by redundancy of metabolic pathways [33]. These attributes make spermatozoa a suitable and robust primary cell type for the establishment of a cellular motility assay. Our model primarily uses semen from healthy men. However, testing spermatozoa from diseased or exposed patients by other reasons than uremia is also possible. Furthermore, the progressive motility of spermatozoa also depends on the seminal fluid, including nutritional substances and functional prostasomes. In the absence of prostasomes, a local non-progressive tail motility occurs [21]. We reason that our model can also be used to analyze the effects of substances on the separate compounds of semen (i.e., spermatozoa versus prostasomes). Additionally, the study of the functional responses to the uremic milieu can vary dependent on the cell type, however, it should be strongly connected with a holistic functionality.

In our clinical pilot study, we show that hemodialysis reduces the mitochondrial toxicity in the serum of the patients [6]. This prompts further clinical investigations on dialysis membrane efficacy with regards to the removal of uremic compounds that can be indirectly analyzed by our model. For this, one must take into consideration that there is a wide range of dialyzers on the market. Dialyzer membranes harbor different capacities of solute removal, e.g., high-flux membrane or superflux membranes remove middle molecule and protein-bound substances to a higher extent as compared to low-flux membranes [34]. In addition, the principle of convection, and/or diffusion, results in the differentiation of the toxin removal capacity. Prolonged dialysis may enable more efficient removal of larger and protein-bound substances, not only from the intravascular but also from the extracellular and intracellular space [35]. Tailorizing hemodialysis application based on our cellular model may add knowledge, not only to the individual level, but also to the practical application of dialysis techniques.

We envision that cellular models of uremic toxicity will level-up with the concrete clinical situation of chronic kidney disease patients. The key aspects for the establishment that are presented here may enable us to build cell models suitable for uremic toxicity research.
